# A Markov chain Monte Carlo (MCMC) methodology with bootstrap percentile estimates for predicting presidential election results in Ghana

**DOI:** 10.1186/s40064-015-1310-2

**Published:** 2015-09-18

**Authors:** Ezekiel N. N. Nortey, Theophilus Ansah-Narh, Richard Asah-Asante, Richard Minkah

**Affiliations:** Department of Statistics, University of Ghana, Box LG 115, Legon, Ghana; Ghana Space Science and Technology Institute (GSSTI), Ghana Atomic Energy Commission (GAEC), Box AE 1, Atomic Kwabenya, Ghana; Department of Political Science, University of Ghana, Legon, Ghana

**Keywords:** Markov chains, Stochastic matrix, Elections, Forecasting, NDC, NPP, Ghana

## Abstract

Although, there exists numerous literature on the procedure for forecasting or predicting election results, in Ghana only opinion poll strategies have been used. To fill this gap, the paper develops Markov chain models for forecasting the 2016 presidential election results at the Regional, Zonal (i.e. Savannah, Coastal and Forest) and the National levels using past presidential election results of Ghana. The methodology develops a model for prediction of the 2016 presidential election results in Ghana using the Markov chains Monte Carlo (MCMC) methodology with bootstrap estimates. The results were that the ruling NDC may marginally win the 2016 Presidential Elections but would not obtain the more than 50 % votes to be declared an outright winner. This means that there is going to be a run-off election between the two giant political parties: the ruling NDC and the major opposition party, NPP. The prediction for the 2016 Presidential run-off election between the NDC and the NPP was rather in favour of the major opposition party, the NPP with a little over the 50 % votes obtained.

## Background

The prime concern for any political party is to map up strategies that would aid them to win an election particularly, the presidential election. This is of key interest to political analysts and the mass media as they would like to discuss and compare parties’ campaign strategies. There is the need therefore to study these political strategies and come up with a mathematical model to predict future elections. Most researchers (Wang et al. 2014; Boon 2012; Campbell and Lewis-Beck 2008) have published papers on election forecasting using opinion polls but not on Markov chain Monte Carlo (MCMC) approach. This research is motivated in introducing this statistical technique to predict the election results in Ghana.

Elections in Ghana can be classified as a random process and similar to the incremental methods, the knowledge of outcomes of previous elections can be used for predictions of future elections. In probability theory, Markov chains are an important type of processes used to study experiments in which the outcomes can be affected by the outcomes of all previous experiments. What is more important about Markov chains is that the outcome of an experiment depends only on the previous experiment. The Ghana Presidential elections from the fourth republic often appear to “flip-flop” after two terms (i.e. a National Democratic Congress (NDC) candidate will win two terms and a National Patriotic Party (NPP) candidate will win the next two terms). MCs should therefore be a useful tool for predicting election results. However, the large literature on methods of predicting election results does not include Markov chain (MC) models in Ghana. One can find the studies on the US presidential elections and the British elections using Markov chains (see for example Wagner 2012; Certin and Bentli 2013).

This paper uses Markov chains generated from previous election data to predict the 2016 presidential elections in Ghana. Confidence intervals for these predictions are obtained from bootstrap percentiles.

### Electoral history of Ghana

The country Ghana which was formerly called the Gold Coast came into existence after so many years of being under the British colony and German-Togo land territory. In 1957, Ghana gained independence under the leadership of Osagyefo Dr. Kwame Nkrumah and became the first West African country to have won freedom from its colonial masters. For over a decade, in 1966–69, 1972–79 and 1981–92 respectively (Asante and Gyimah-Boadi 2004) there had been numerous coup d’états which had affected the socio-economic processes of the new born country Ghana.

When Ft. Lt. Jerry John Rawlings took over power in 1981 (Rothschild 1985), he banned political parties until 1992 (Handley 2008) when he lifted the ban and restored the country Ghana to multiparty democracy and also introduced a new constitution. He later formed a new party called the National Democratic Congress (NDC) and was voted into power in 1992 and 1996 elections (Bimpong-Buta 2005).

After his 2nd term, a new opposition party by then known as the National Patriotic Party (NPP) was formed under the Dankwa-Busia tradition (Ayee 2009) and led by John Agyekum Kuffour also won for two terms, in 2000 and 2004 elections.

The NDC again is in its 2nd term (i.e. 2008-date) for the 2nd time and is currently led by John Dramani Mahama

Since the introduction of the new constitution by Rawlings in 1992, voting patterns have been swindling and that’s why it is of key interest to researchers, political analysts and mass media as a whole, to find answers to why this phenomenon.

**Fig. 1:**
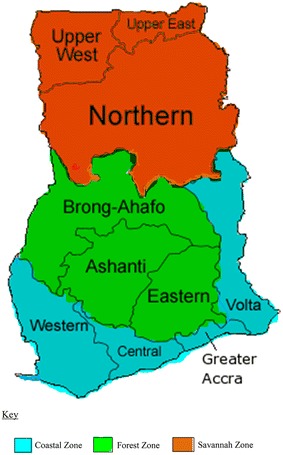
A map of Ghana showing the three zones

Ghana as displayed in Fig. 1 is spatially divided into three ecological zones, namely: the Savannah belt that consists of the Northern, Upper East and Upper West regions; the Forest or Middle belt consisting of Ashanti, Brong Ahafo and Eastern regions with the largest representation of the Akans and finally the Coastal belt which consists of the Western, Central, Greater Accra an Volta regions. It is believed that voting is actually characterized by ethnic sentiments and thus the study would want to find out if predicted results of the 2016 elections really follow that assertion.

## Markov chains

Let *X* = {*X*_0_, *X*_1_,…} be a sequence of random variables taking values in some countable set *S* = {*s*_1_, *s*_2_,…} referred to as *state space*. The sequence {*X*_0_, *X*_1_,…}is called a Markov chain if

1 $$P\left( {X_{k} = j\left| {X_{0} = x_{0} , \ldots ,\,X_{k - 1} = } \right.i} \right) = P\left( {X_{k} = j\left| {X_{k - 1} = i} \right.} \right)$$

for all *k* ≥ 1 and *x*_0_,…, *i*, *j* in *S*. In addition, if

2 $$P\left( {X_{k} = j\left| {X_{k - 1} = i} \right.} \right) = p_{ij} ,$$

then the Markov chain is homogeneous. Here, *p*_*ij*_ in Eq. (2) is referred to as the matrix of transition probabilities and it satisfies the following conditions:

3 $$0 \le p_{ij} \le 1$$

and

4 $$\sum\limits_{j} {p_{ij} = 1}$$

Each transition is called a step. Any matrix satisfying Eqs. (2), (3) and (4) is referred to as a stochastic matrix. In addition if $\sum\limits_{i} {p_{ij} = 1}$ then it is called a doubly stochastic matrix.

The first-order difference equation of a MC is expressed as

5 $$\phi_{r + 1} = {\mathbf{P}}\phi_{r} ,\,\,\,r = 1,\,\,2,\, \ldots ,\,\,m$$

where **P** is an m-by-m square matrix.

### Theorem 1

Let **P** be a matrix of transition probabilities of a Markov chain. The *ij*th element $p_{{_{ij} }}^{n}$ of the matrix **P**^*n*^ is the given probability that the Markov chain starting in state *s*_*i*_ will transition to state *s*_*j*_ after *n*-steps.

If *p*_*ij*_ is regular, then there is a unique vector *ϕ*_*r*_ such that, for any probability vector *ϕ*_0_ and for large values of *r*,

6 $$\mathop {\lim }\limits_{r \to \infty } \phi_{r + 1} = {\mathbf{P}}^{r} \phi_{0} .$$

Here the vector *ϕ*_*r*_ in Eq. (6) is called equilibrium or an ergodic vector of the MC. Therefore, we can compute probability vectors given that the transition matrix and the original probability vector are known (Lay 2011; Lial et al. 2012).

## Methodology

In Ghana, the Presidential election results are determined by the Electoral Commission (EC) and the elections are carried out at various constituencies in each Region. In this paper, the Upper East, Upper West and Northern regions form the Savannah Zone; Brong-Ahafo, Ashanti and Eastern regions form the Forest Zone; and Western, Central, Greater Accra and Volta regions form the Coastal Zone. In the Ghana Presidential elections, each candidate receives a certain number of votes and the candidate with more than 50 % of the total valid votes casted wins the presidential election in Ghana. Otherwise, a run-off election is organized for the two topmost candidates.

We used the 1992–2008 Presidential election results to generate a stochastic matrix and the 2012 Presidential results as the probability vector to predict the 2016 Presidential election results. Following the methodology of Wagner (2012), the transition probability matrices are created from the previous election results as depicted in Table 1.

**Table 1:** National presidential election (PE) votes for the period 1992–2012

No.	Year	NDC	NPP	Other	Rejected votes
1	1992	58.4	30.3	11.30	0^b^
2	1996	57.4	39.7	1.37	1.53
3	2000	44.5	48.17	5.53	1.80
4	2000^a^	43.10	56.90	0	0
5	2004	44.64	52.45	0.78	2.13
6	2008	47.92	49.13	0.55	2.4
7	2008^a^	50.23	49.77	0	0
8	2012	50.70	47.74	1.21	0.35

Source: Ghana electoral commission certified results

^a^Indicates run-off votes

^b^Indicate a very negligible proportion close to zero

We let *ϕ*_*i*_, *i* = 1, 2,…, 8 represent the presidential election results for 1992, 1996,…, 2012. Thus, we have:

7 $$\left. {\begin{array}{*{20}c} {\phi_{1} = \left( {0.5840\,\,,\,\,\,0.3030\,\,,\,\,\,0.1130\,\,\,,\,\,\,0.0000} \right)^{\prime } } \\ {\phi_{2} = \left( {0.5740\,\,\,,\,\,\,0.3970\,\,\,,\,\,\,0.0137\,\,,\,\,\,0.0153} \right)^{\prime } } \\ {\phi_{3} = \left( {0.4450\,\,\,,\,\,\,0.4817\,\,,\,\,\,0.0553\,\,\,,\,\,\,0.0180} \right)^{\prime } } \\ {\phi_{4} = \left( {0.4310\,\,\,,\,\,\,0.5690\,\,,\,\,\,0.0000\,\,,\,\,\,0.0000} \right)^{\prime } } \\ {\phi_{5} = \left( {0.4464\,\,\,,\,\,\,0.5245\,\,\,,\,\,\,0.0078\,\,\,,\,\,\,0.0213} \right)^{\prime } } \\ {\phi_{6} = \left( {0.4792\,\,\,,\,\,0.4913\,\,\,,\,\,\,0.0055\,\,\,,\,\,\,0.0240} \right)^{\prime } } \\ {\phi_{7} = \left( {0.5023\,\,\,,\,\,0.4977\,\,\,,\,\,\,0.0000\,\,\,,\,\,\,0.0000} \right)^{\prime } } \\ {\phi_{8} = \left( {0.5070\,\,\,,\,\,\,0.4774\,\,\,,\,\,\,0.0121\,\,\,,\,\,\,0.0035} \right)^{\prime } } \\ \end{array} } \right\}$$

The stochastic matrix for the model is thus obtained by averaging the transformation of the previous election results. This is the so-called Average Transformation Method (ATM) of Wagner (2012). Let *L*_*i*_, *i* = 1,…, 7 be the transformation matrix from *i*th to the (*i* + 1)th election results such that *L*_*i*_*ϕ*_*i*_ = *ϕ*_*i*+1_. For instance, *L*_1_is the transformation matrix of the Presidential Elections results from 1992 to 1996 is given by

8 $$L_{1} = \begin{array}{*{20}c} {\begin{array}{*{20}c} {{\text{NDC}}} & {{\text{NPP}}} & {\text{O}} & {\text{R}} \\ \end{array} } & {} \\ {\left[ {\begin{array}{*{20}c} {l_{{11}} } & {l_{{12}} } & {l_{{13}} } & {l_{{14}} } \\ {l_{{21}} } & {l_{{22}} } & {l_{{23}} } & {l_{{24}} } \\ {l_{{31}} } & {l_{{32}} } & {l_{{33}} } & {l_{{34}} } \\ {l_{{41}} } & {l_{{42}} } & {l_{{43}} } & {l_{{44}} } \\ \end{array} } \right]} & {\begin{array}{*{20}l} {{\text{NDC}}} \\ {{\text{NPP}}} \\ {\text{O}} \\ {\text{R}} \\ \end{array} } \\ \end{array}$$

where, O and R are Other parties and Rejected votes respectively. Here, *L*_1_ is unknown but the probability vectors for the 1992 and 1996 elections are known and hence from Eq. (5), we have

9 $$\left[ {\begin{array}{*{20}c} {l_{11} } & {l_{12} } & {l_{13} } & {l_{14} } \\ {l_{21} } & {l_{22} } & {l_{23} } & {l_{24} } \\ {l_{31} } & {l_{32} } & {l_{33} } & {l_{34} } \\ {l_{41} } & {l_{42} } & {l_{43} } & {l_{44} } \\ \end{array} } \right]\left( \begin{aligned} 0.5840 \hfill \\ 0.3030 \hfill \\ 0.1130 \hfill \\ 0 \hfill \\ \end{aligned} \right) = \left( \begin{aligned} 0.5740 \hfill \\ 0.3970 \hfill \\ 0.0137 \hfill \\ 0.0153 \hfill \\ \end{aligned} \right)$$

where, *l*_11_ is the percentage of people who voted for NDC in the 1992 PE that also voted for the same party in the 1996 PE. Similar, explanations holds for *l*_*ij*_, ∀*i*, *j* = 1, 2, 3, 4.

For the use of MC analysis, the following assumptions were made:

1. Everyone who voted in the preceding election year voted in the following election year.
2. There is an equal probability for voting for another party in the following election year provided you did not vote for these parties in the preceding election year.
3. Other parties which did not take part in run-off elections were recorded zero.
4. There is no rejected votes in all run-off elections

Based on the first assumption,

$$l_{11} = \frac{0.5740}{0.5840} = 0.9829,$$

and

$$l_{12} = l_{13} =l_{14}=0.0057.$$

Similarly, the percentage of other political parties *l*_33_ = 0.0137/0.01370.1130.0.1130 = 0.1212 and *l*_31_ = *l*_32_ = *l*_34_ = 0.2929. However, the percentage of NPP votes increased from 1992 to 1996, so we have *l*_22_ = 1 and *l*_21_ = *l*_23_ = *l*_24_ = 0.

In addition *l*_44_ = 1 and *l*_41_ = *l*_42_ = *l*_43_ = 0.

Therefore, as specified in Eq. (8), we have:

$$L_{1} = \left( {\begin{array}{*{20}c} {0.9829} & {0.0057} & {0.0057} & {0.0057} \\ 0 & 1 & 0 & 0 \\ {0.2929} & {0.2929} & {0.1212} & {0.2929} \\ 0 & 0 & 0 & 1 \\ \end{array} } \right)$$

The same procedure is followed to obtain the other transformation matrices *L*_2_…., *L*_7_. The average of the transition matrices are obtained as ${\mathbf{P}} = 7^{ - 1} \sum\limits_{i = 1}^{7} {L_{i} }$.

Using the steady state property of Eq. (5), we obtain the following results as shown in Table 2.

**Table 2:** Predicted 2016 presidential elections with bootstrap standard errors

Year	NDC	NPP	Other	Rejected
Percentage	48.70	47.80	1.80	1.60
SE	4.80	6.4	0.26	0.80

Source: author’s computation

Since no candidate is expected to obtain more than 50 % in the 2016 Presidential votes by the model results: there will be no clear winner in the 2016 first round elections. Hence, a run-off vote between the two dominant parties i.e. the NDC and the NPP.

**Fig. 2:**
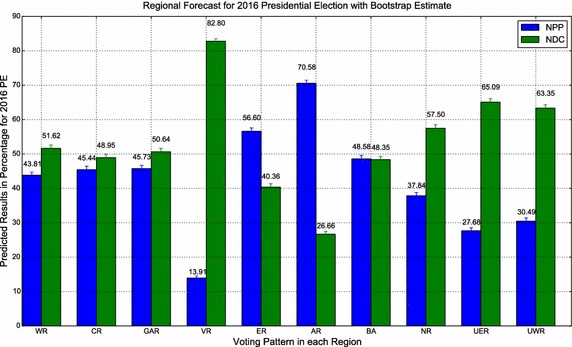
Regional forecasts for 2016 Presidential Election with bootstrap estimates

**Fig. 3:**
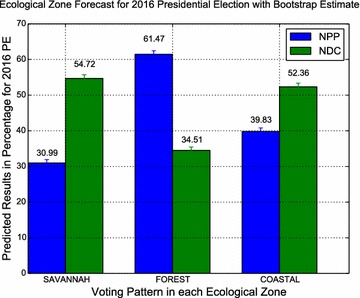
Ecological Zone Forecasts for 2016 Presidential Election with Bootstrap Estimates

To model this, we follow assumptions 3 and 4 to modify Table 1 as follows:

Applying the procedure to the generated observations in Table 3 yields the predicted values as shown in Table 4. Figures 2 and 3 display respectively, the regional and ecological zone forecasts 2016 Presidential Election with Bootstrap estimates.

**Table 3:** Suggested national presidential run-off votes

Year	% NDC	% NPP	% Other	% Rejected votes
1992^a^	64.05	35.95	0	0
1996^a^	58.85	41.15	0	0
2000^a^	49.17	51.83	0	0
2000^b^	43.10	56.90	0	0
2004^a^	46.10	53.90	0	0
2008^a^	49.40	50.60	0	0
2008^b^	50.23	49.77	0	0
2012^a^	51.48	48.52	0	0

Source: authors’ computation

^a^Winner declared in first round votes

^b^ Run-off results

**Table 4:** Predicted 2016 presidential run-off election results with bootstrap standard errors

	NDC	NPP
Percentage	48.30	51.70
Bootstrap SE	5.90	5.90

Source: author’s computation

Similarly the same methodology was applied to the regional and ecological Presidential Election results to predict the run-off results in 2016. The results are as shown below:

Table 5 shows the model’s predictions for the regional presidential election results for the 2016 presidential elections. The results show that the NDC is the popular choice of voters in the Western (50.64 %), Greater Accra (50.64 %), Volta (82.8 %), Northern (57.5 %), Upper East (65.09 %), and Upper West (63.35 %) whereas the NPP is popular in the Eastern (50.6 %) and Ashanti (70.58 %) regions.

**Table 5:** Forecasted regional presidential election results for 2016

Region	NDC		NPP		Other	Rejected votes	
	Main (%)	Run-off (%)	Main (%)	Run-off (%)	Main (%)	Main (%)	Run-off (%)
National	48.70	48.30	47.80	51.70	1.80	1.60	–
Western	51.62	53.27	43.81	46.73	2.21	2.36	–
Central	48.95	50.95	45.44	49.05	2.55	3.08	–
Greater Accra	50.64	52.44	45.73	47.56	1.73	1.90	–
Volta	82.80	83.77	13.91	16.23	1.33	1.96	–
Eastern	40.36	41.21	50.60	58.79	1.47	1.56	–
Ashanti	26.66	27.63	70.58	72.37	1.29	1.48	–
Brong Ahafo	48.35	49.38	48.58	50.62	1.56	1.51	–
Northern	57.50	60.00	37.84	40.00	2.14	2.52	–
Upper East	65.09	68.93	27.68	31.07	3.84	3.39	–
Upper West	63.35	63.46	30.49	35.54	2.82	3.35	–

Source: author’s computation

– Rejected votes are not factored into the computations of the probabilities

The prediction of the Ecological zone presidential election results are presented in Table 6. The NDC has over 50 % of valid votes from the Savannah and Coastal belts whereas the NPP, their closest oponents remain the toast of the forest belt.

**Table 6:** Forecasted ecological zone presidential election results for 2016

Ecological	NDC		NPP		Other	Rejected votes	
	Main election (%)	Round-off (%)	Main election (%)	Round-off (%)	Main election (%)	Main election (%)	Run-off (%)
National	49.72	48.30	47.52	51.70	1.80	0.96	–
Savannah	54.72	62.01	30.99	37.99	2.72	11.57	–
Forest zone	34.51	38.42	61.47	61.58	1.96	2.06	–
Coastal zone	52.36	52.66	39.83	47.34	3.89	3.95	–

Source: author’s computation

### Forecasting the regional presidential votes for 2016

#### Western region

$$\left[ { 0. 5 4 4 2 { }\,\, 0. 4 4 1 2 { }\,\, 0. 0 0 4 8 { }\,\, 0. 0 0 9 8} \right]\left[ \begin{aligned} 0. 9 3 3 5 { 0} . 0 2 2 2 { 0} . 0 2 2 2 { 0} . 0 2 2 2\hfill \\ 0. 0 1 3 3 { 0} . 9 6 0 1 { 0} . 0 1 3 3 { 0} . 0 1 3 3\hfill \\ 0. 1 5 5 0 { 0} . 1 5 5 0 { 0} . 5 3 4 9 { 0} . 1 5 5 0\hfill \\ 0. 1 6 6 7 { 0} . 1 6 6 7 { 0} . 1 6 6 7 { 0} . 5 0 0 0\hfill \\ \end{aligned} \right]$$

which equals $$\left[ {0. 5 1 6 2 { }\,\,0. 4 3 8 1 { }\,0.\,0 2 2 1 { }\,\, 0. 0 2 3 6} \right]$$

#### Central region

$$\left[ { 0. 5 2 1 2 { }\,\, 0. 4 5 5 3\,\,{ 0} . 0 0 2 5\,\,{ 0} . 0 2 1 0} \right]\left[ \begin{aligned} 0. 9 2 0 1 \quad{ 0} . 0 2 6 6 \quad{ 0} . 0 2 6 6 \quad{ 0} . 0 2 6 6\hfill \\ 0. 0 1 3 6 \quad{ 0} . 9 5 9 2\quad { 0} . 0 1 3 6\quad { 0} . 0 1 3 6\hfill \\ 0. 1 1 1 1\quad { 0} . 1 1 1 1\quad { 0} . 6 6 6 7\quad { 0} . 1 1 1 1\hfill \\ 0. 1 6 6 7\quad { 0} . 1 6 6 7\quad { 0} . 1 6 6 7\quad { 0} . 5 0 0 0\hfill \\ \end{aligned} \right]$$

which equals $$\left[ {0. 4 8 9 5 { }\,\,0. 4 5 4 4 { }\,0.0 2 5 2 { }\,\,0.0 30 8} \right]$$

#### Greater Accra region

$$\left[ { 0. 5 2 1 1 { }\,\, 0. 4 7 1 1 { }\,\, 0. 0 0 1 7 { }\,\, 0. 0 0 6 1} \right]\left[ \begin{array}{llll}0. 9 5 5 0& 0 . 0 1 5 0&0. 0 1 5 0&0. 0 1 5 0\\ 0. 0 1 6 1&0 . 9 5 1 6&0 . 0 1 6 1 & 0 . 0 1 6 1\\ 0. 1 3 5 9& 0 . 1 3 5 9& 0 . 5 9 2 3 &0 . 1 3 5 9\\ 0. 1 5 1 9 &0 . 1 5 1 9&0 . 1 5 1 9&0. 5 4 4 4\\ \end{array} \right]$$

which equals $$\left[ {0. 50 6 4 { }\,0. 4 5 7 3 { }\,0.0 1 7 3 { }\,\,0.0 1 90} \right]$$

#### Volta region

$$\left[ { 0. 8 4 4 6 { }\,\, 0. 1 2 9 2\,\,{ 0} . 0 0 4 1\,\,{ 0} . 0 2 2 1} \right]\left[ \begin{aligned} { 0} . 9 7 4 7\quad { 0} . 0 0 8 4\quad { 0} . 0 0 8 4\quad { 0} . 0 0 8 4\hfill \\ 0. 0 0 3 9\quad { 0} . 9 8 8 4\quad { 0} . 0 0 3 9\quad { 0} . 0 0 3 9\hfill \\ 0. 1 6 2 5 \quad { 0} . 1 6 2 5\quad { 0} . 5 1 2 4\quad { 0} . 1 6 2 5\hfill \\ 0. 1 6 3 2\quad { 0} . 1 6 3 2\quad { 0} . 1 6 3 2\quad { 0} . 5 1 0 4\hfill \\ \end{aligned} \right]$$

which equals $$\left[ {0. 40 3 6 { }\,0. 5 6 60 \, \,0.0 1 4 7 { }\,\,0.0 1 5 6} \right]$$

#### Eastern region

$$\left[ { 0. 4 2 6 1 { }\,\, 0. 5 6 3 0 { }\,\, 0. 0 0 2 0 { }\,\, 0. 0 0 8 9} \right]\left[ \begin{aligned} { 0} . 9 3 6 2 \quad { 0} . 0 2 1 3\quad { 0} . 0 2 1 3\quad { 0} . 0 2 1 3\hfill \\ 0. 0 0 4 8 \quad { 0} . 9 8 5 7\quad { 0} . 0 0 4 8\quad { 0} . 0 0 4 8\hfill \\ 0. 1 3 1 6\quad { 0} . 1 3 1 6\quad { 0} . 6 0 5 3\quad { 0} . 1 3 1 6\hfill \\ 0. 1 9 9 4\quad { 0} . 1 9 9 4 \quad { 0} . 1 9 9 4\quad { 0} . 4 0 1 7\hfill \\ \end{aligned} \right]$$

which equals $$\left[ {0. 8 2 80 \, \,0. 1 3 9 1 { }\,0.0 1 3 3 { }\,\,0.0 1 9 6} \right]$$

#### Ashanti region

$$\left[ { 0. 2 8 3 5 { }\,\, 0. 7 0 8 0\,\,{ 0} . 0 0 1 2\,\,{ 0} . 0 0 7 3} \right]\left[ \begin{aligned} { 0} . 9 2 2 5 \quad { 0} . 0 2 5 8 \quad { 0} . 0 2 5 8 \quad { 0} . 0 2 5 8\hfill \\ 0. 0 0 5 1\quad { 0} . 9 8 4 6\quad { 0} . 0 0 5 1\quad { 0} . 0 0 5 1\hfill \\ 0. 1 4 7 2 \quad { 0} . 1 4 7 2\quad { 0} . 5 5 8 4\quad { 0} . 1 4 7 2\hfill \\ 0. 1 6 6 7 \quad { 0} . 1 6 6 7\quad { 0} . 1 6 6 7\quad { 0} . 5 0 0 0\hfill \\ \end{aligned} \right]$$

which equals $$\left[ {0. 2 6 6 6 { }\,0. 70 5 8 { }\,0.0 1 2 9 { }\,\,0.0 1 4 8} \right]$$

#### Brong Ahafo region

$$\left[ { 0. 5 0 7 4 { }\,\, 0. 4 9 0 0\,\,{ 0} . 0 0 2 6\,\,{ 0} . 0 0 0} \right]\left[ \begin{aligned} { 0} . 9 4 2 4\quad { 0} . 0 1 9 2\quad { 0} . 0 1 9 2\quad { 0} . 0 1 9 2\hfill \\ 0. 0 0 9 8 \quad { 0} . 9 7 0 6 \quad { 0} . 0 0 9 8 \quad { 0} . 0 0 9 8\hfill \\ 0. 2 0 2 6 \quad { 0} . 2 0 2 6\quad { 0} . 3 9 2 3\quad { 0} . 2 0 2 6\hfill \\ 0. 1 6 6 7\quad { 0} . 1 6 6 7\quad { 0} . 1 6 6 7\quad { 0} . 5 0 0 0\hfill \\ \end{aligned} \right]$$

which equals $$\left[ {0. 4 8 3 5 { }\,0. 4 8 5 8 { }\,0.0 1 5 6 { }\,\,0.0 1 5 1} \right]$$

#### Northern region

$$\left[ { 0. 5 8 2 2\,\,{ 0} . 3 9 1 1\,\,{ 0} . 0 0 7 5\,\,{ 0} . 0 1 9 2} \right]\left[ \begin{aligned} 0. 9 6 6 5\quad { 0} . 0 1 1 2\quad { 0} . 0 1 1 2\quad { 0} . 0 1 1 2\hfill \\ 0. 0 2 0 2 \quad { 0} . 9 3 9 5\quad { 0} . 0 2 0 2 \quad { 0} . 0 2 0 2\hfill \\ 0. 1 6 5 0 \quad { 0} . 1 6 5 0\quad { 0} . 5 0 5 0\quad { 0} . 1 6 5 0\hfill \\ 0. 1 6 6 7 \quad { 0} . 1 6 6 7\quad { 0} . 1 6 6 7\quad { 0} . 5 0 0 0\hfill \\ \end{aligned} \right]$$

which equals $$\left[ {0. 5 7 50 \, \,0. 3 7 8 4 { }\,0.0 2 1 4 { }\,0.0 2 5 2} \right]$$

#### Upper east

$$\left[ { 0. 6 6 4 4\,\,{ 0} . 2 9 2 9\,\,{ 0} . 0 2 2 3\,\,{ 0} . 0 2 0 4} \right]\left[ \begin{aligned} 0. 9 4 9 6\quad { 0} . 0 1 6 8\quad { 0} . 0 1 6 8 \quad { 0} . 0 1 6 8\hfill \\ 0. 0 4 0 4\quad { 0} . 8 7 8 8 \quad { 0} . 0 4 0 4 \quad { 0} . 0 4 0 4\hfill \\ 0. 1 6 9 8\quad { 0} . 1 6 9 8\quad { 0} . 4 9 0 5\quad { 0} . 1 6 9 8\hfill \\ 0. 2 1 7 3\quad { 0} . 2 1 7 3 \quad { 0} . 2 1 7 3\quad { 0} . 3 4 8 1\hfill \\ \end{aligned} \right]$$

which equals $$\left[ {0. 6 50 9 { }\,0. 2 7 6 8 { }\,0.0 3 8 4 { }\,0.0 3 3 9} \right]$$

#### Upper West

$$\left[ { 0. 6 5 5 4\,\,{ 0} . 2 9 2 6\,\,{ 0} . 0 2 0 1\,\,{ 0} . 0 3 1 9} \right]\left[ \begin{aligned} 0. 9 4 9 5\quad { 0} . 0 1 6 8 \quad { 0} . 0 1 6 8\quad { 0} . 0 1 6 8\hfill \\ 0. 0 0 8 5\quad { 0} . 9 7 4 5 \quad { 0} . 0 0 8 5\quad { 0} . 0 0 8 5\hfill \\ 0. 1 7 5 9\quad { 0} . 1 7 5 9\quad { 0} . 4 7 2 2 \quad { 0} . 1 7 5 9\hfill \\ 0. 1 6 1 9\quad { 0} . 1 6 1 9\quad { 0} . 1 6 1 9\quad { 0} . 5 1 4 2\hfill \\ \end{aligned} \right]$$

which equals $$\left[ {0. 6 3 3 5 { }\,0. 30 4 9 { }\,0.0 2 8 2 { }\,0.0 3 3 5} \right]$$

### Forecasting ecological zone for presidential votes

#### Savannah zone

$$\left[ {0. 5 5 3 8 { }0. 3 1 5 5 { }0.0 1 2 0 { }0. 1 1 8 8} \right]\left[ \begin{aligned} 0. 9 5 9 0\quad { 0} . 0 1 3 7\quad { 0} . 0 1 3 7\quad { 0} . 0 1 3 7\hfill \\ 0. 0 2 3 2 \quad { 0} . 9 3 0 5\quad { 0} . 0 2 3 2\quad { 0} . 0 2 3 2\hfill \\ 0. 1 7 7 4 \quad { 0} . 1 7 7 4 \quad { 0} . 4 6 7 7\quad { 0} . 1 7 7 4\hfill \\ 0. 0 5 6 2\quad { 0} . 0 5 6 2\quad { 0} . 0 5 6 2\quad { 0} . 8 3 1 5\hfill \\ \end{aligned} \right]$$

which equals $$\left[ {0. 5 4 7 2 { }\,0. 30 9 9 { }\,0.0 2 7 2 { }\,0. 1 1 5 8} \right]$$

#### Forest zone

$$\left[ {0. 3 7 5 0\,\, \, 0. 6 1 9 6\,\, \, 0.00 1 9\,\, \, 0.00 3 6} \right]\left[ \begin{aligned} 0. 9 0 2 3 \quad { 0} . 0 3 2 6\quad { 0} . 0 3 2 6 \quad { 0} . 0 3 2 6\hfill \\ 0. 0 0 9 6 \quad { 0} . 9 7 1 1\quad { 0} . 0 0 9 6\quad { 0} . 0 0 9 6\hfill \\ 0. 1 7 0 9\quad { 0} . 1 7 0 9\quad { 0} . 4 8 7 2 \quad { 0} . 1 7 0 9\hfill \\ 0. 1 3 5 1\quad { 0} . 1 3 5 1\quad { 0} . 1 3 5 1\quad { 0} . 5 9 4 8\hfill \\ \end{aligned} \right]$$

which equals $$\left[ {0. 3 4 5 1 { }\,0. 6 1 4 7 { }\,0.0 1 9 6 { }\,0.0 20 6} \right]$$

#### Coastal zone

$$\left[ {0. 5 8 7 6\,\, \, 0. 40 6 4\,\, \, 0.00 2 9\,\, \, 0.00 3 1} \right]\left[ \begin{aligned} 0. 8 7 0 4\quad { 0} . 0 4 3 2\quad { 0} . 0 4 3 2 \quad { 0} . 0 4 3 2\hfill \\ 0. 0 2 8 1\quad { 0} . 9 1 5 8\quad { 0} . 0 2 8 1 \quad { 0} . 0 2 8 1\hfill \\ 0. 1 3 5 5 \quad { 0} . 1 3 5 5\quad { 0} . 5 9 3 6\quad { 0} . 1 3 5 5\hfill \\ 0. 1 2 4 1\quad { 0} . 1 2 4 1\quad { 0} . 1 2 4 1\quad { 0} . 6 2 7 6\hfill \\ \end{aligned} \right]$$

which equals $$\left[ {0. 5 2 3 6 { }\,0. 3 9 8 3 { }\,0.0 3 8 9 { }\,0.0 3 9 1} \right]$$

## Conclusion

The model used in this study predicted the party that will win the 2016 PE with NDC having 49.72 %, NPP (47.52 %) and Other parties and Rejected votes having 1.8 and 0.96 %. The overall average error in this prediction was estimated as ≈2.4 %. This was determined by finding the absolute percentage differences between the predicted and the actual results for previous elections.

It is evidently clear that both NPP and NDC have approximately 47 % of loyal voters who would always vote for these parties on any day and any time. Therefore with more education on how to reduce rejected votes, certainly would show a significant effect in the 2016 PE. Thus, the party that would channel lots of resources into voter education could sway the results in its favour.

A further study on this research is to also use other sophisticated mathematical models like Bayesian Estimation to compare the results of this method.
